# Reaching a consensus on a patellofemoral pain syndrome self-management programme for recreational cyclists in Saudi Arabia: A modified Delphi study

**DOI:** 10.4102/sajp.v82i1.2271

**Published:** 2026-02-28

**Authors:** Ameen Masoudi, Bashir Bello, Nomzamo Chemane, Ushotanefe Useh, Nontembiso Magida

**Affiliations:** 1 Department of Physiotherapy, School of Health Sciences, College of Health Sciences, University of KwaZulu-Natal, Durban, South Africa; 2 Department of Physiotherapy, Faculty of Allied Health Sciences, Bayero University, Kano, Nigeria; 3 Lifestyle Diseases Research Entity, Faculty of Health Sciences, North-West University, Mahikeng, South Africa; 4 Department of Physiotherapy, School of Health Care Sciences, Faculty of Health Sciences, University of Pretoria, Pretoria, South Africa

**Keywords:** patellofemoral pain syndrome, self-management, cycling, Delphi method, rehabilitation

## Abstract

**Background:**

Recreational cycling is increasingly popular in Al Madinah, Saudi Arabia. However, many cyclists lack structured self-management programmes (SMPs) to address patellofemoral pain syndrome (PFPS), a common overuse injury that significantly affects knee function and cycling performance. Despite the growing interest in cycling, a critical gap remains in culturally relevant, evidence-based SMP.

**Objectives:**

Our study aimed to develop expert consensus on the essential components of SMP for recreational cyclists with PFPS.

**Method:**

Our study employed a two-round modified Delphi to develop expert consensus on the components of an SMP for recreational cyclists with PFPS. A panel of 25 experts, comprising physiotherapists, biokinetists, sports medicine professionals and exercise physiologists, participated in Round 1, with 19 experts (76% retention) completing Round 2. The process involved rating proposed programme objectives, principles, outcome measures and pain relief strategies using a four-point Likert scale. Consensus was defined as ≥ 70% of respondents selecting either ‘disagree’ to ‘strongly agree’.

**Results:**

Consensus was reached on strengthening exercises (88%), evidence-based practice (84%), pain education (84%), PFPS education (92%) and knee pain management (92%). Round 2 results were streamlined and ranked according to pain, function, adherence, cultural resources and engagement supports, following clarifications and the addition of new items from experts.

**Conclusion:**

Our study establishes an evidence-based, region-specific framework for self-managing PFPS among recreational cyclists.

**Clinical implications:**

The proposed programme is designed to enhance adherence, reduce the risk of injury and improve cycling performance. Future research should evaluate the effectiveness of this approach, its scalability, outcomes and integration into national sports and rehabilitation strategies.

## Introduction

Recreational cycling has surged in popularity across Saudi Arabia, attracting individuals for its well-documented health benefits, social engagement and accessibility (Althunyan, Darwish & Wahab 2017). Regular cycling improves cardiovascular fitness, boosts muscular endurance and enhances psychological well-being (Nordengen et al. 2019). Studies demonstrate its role in reducing the risks of chronic diseases, such as obesity and diabetes, while promoting mental health through stress reduction (Götschi, Garrard & Giles-Corti 2016). In Saudi Arabia, this rise aligns with a growing interest in physical activity amid a historically sedentary culture, spurred by government initiatives such as Vision 2030, which promotes healthier lifestyles. However, as participation increases, so do challenges related to injury prevention, training optimisation and self-management practices.

Despite this growth, no evidence-based SMP exists for recreational cyclists in the region, leaving a significant gap in support for this burgeoning community.

Cycling’s demands extend beyond physical benefits. It fosters a sense of community and social belonging, particularly valuable in areas with limited structured sports opportunities (Götschi et al. 2016). In Saudi Arabia, where traditional sports infrastructure has been underdeveloped, cycling offers an accessible entry point for exercise, often requiring minimal equipment beyond a bicycle. While this democratisation of the sport is promising, it also amplifies risks for those unprepared for its physical demands. Despite its advantages, cycling carries inherent risks, particularly for recreational cyclists who lack self-management skills. Overuse injuries, such as patellofemoral pain syndrome (PFPS), improper bike fitting, hydration mismanagement and inadequate recovery are prevalent (Prieto-González et al. 2021). Patellofemoral pain syndrome, a common knee condition among cyclists, is often caused by repetitive stress and poor biomechanics, which can be exacerbated by suboptimal equipment or technique (Althunyan et al. 2017). In Saudi Arabia, these risks are compounded by environmental and infrastructural factors.

Extreme temperatures, frequently exceeding 40°C, challenge hydration and heat management, while limited cycling-friendly infrastructure, such as dedicated lanes or shaded routes, intensifies the potential for injury (Althunyan et al. 2017; Tarawneh & Chowdhury 2018). Urban areas, such as Riyadh and Jeddah, offer some facilities, but rural and desert regions often lack them, forcing cyclists to navigate uneven terrain or busy roads. These conditions underscore the urgent need for a structured SMP tailored to the region’s unique context.

Self-management programmes empower athletes to regulate training loads, mitigate injuries and optimise recovery, a principle well-established in endurance sports (Phillips & Hopkins 2020).

In cycling, where performance and injury risk are tightly linked to self-regulation, such frameworks enhance consistency and longevity (Phillips & Hopkins 2020). Effective programmes typically integrate nutrition planning, strength and conditioning, sleep optimisation and injury prevention strategies, customised to participants’ needs (Masoudi et al. 2025). For instance, strength exercises targeting the quadriceps and hips can reduce the incidence of PFPS, while hydration protocols can mitigate heat-related fatigue (Passigli, Capacci & Volpi 2017). However, without a standardised, culturally relevant programme, Saudi recreational cyclists struggle to adopt these practices, often relying on trial and error or anecdotal advice. This gap is particularly stark given the sport’s physical demands and the region’s environmental stressors, which amplify the consequences of poor preparation.

The efficacy of self-management is well-documented in other endurance disciplines. Studies in running and swimming show reduced injury rates and improved performance through structured self-regulation (Wu et al. 2021). For example, peer-led programmes in running have reduced overuse injuries by 30%, while swimming interventions enhance recovery by focusing on sleep and nutrition (Malachowski 2016). Professional cyclists benefit from sports science support, including coaches, physiotherapists and data-driven training plans. However, recreational cyclists rarely access such resources (Masoudi et al. 2025). Instead, they turn to informal sources, such as online forums or peers, which may lack scientific grounding. This disparity underscores the need for an accessible, evidence-based SMP tailored to Saudi Arabia’s recreational cycling community, thereby bridging the gap between elite and amateur support systems. With notable advancements in Africa and other regions, SMP in physiotherapy is gaining increasing popularity worldwide.

A national survey conducted in Ghana found that more than 60% of physiotherapists used multimodal techniques to treat chronic low back pain, which included massage, electrotherapy, exercises and home advice (Oppong-Yeboah & May 2014). Similar to this, a feasibility study conducted in Ghana demonstrated the potential of an exercise and biopsychosocial education programme guided by a physiotherapist for managing low back pain, highlighting the value of combining education with physical therapy (Motha et al. 2024). According to a study conducted in Ethiopia, many healthcare practitioners faced fragmented information and encountered obstacles, including systemic constraints and a lack of competency, when providing SMP for persistent low back pain (Chala et al. 2022).

Developing such a programme requires a consensus-driven approach to ensure relevance and applicability. The Delphi method, widely used in sports science and healthcare, offers a systematic framework for achieving expert agreement on programme components (Nasa, Jain & Juneja 2021). By synthesising diverse perspectives, including those of sports physiotherapists, specialist musculoskeletal physiotherapists and experienced cyclists, the Delphi method aimed to establish a robust, multidisciplinary foundation (Nasa et al. 2021). This method’s strength lies in its ability to complex, subjective inputs into an integrated strategy, making it ideal for addressing the multifaceted needs of Saudi Arabian cyclists. Previous Delphi studies have successfully shaped interventions in sports medicine, such as injury prevention protocols for runners, demonstrating their utility (Brady 2015). A key advantage of the Delphi method is its iterative nature, which allows for continuous refinement through multiple feedback rounds (Brady 2015). Our study leverages this process to create a comprehensive framework tackling injury prevention, training periodisation, psychological resilience and environmental adaptation. Injury prevention, for instance, might include bike-fitting guidelines to reduce PFPS, while periodisation could balance training intensity with Saudi Arabia’s seasonal heat peaks. Psychological resilience is crucial for maintaining motivation in a challenging environment and can incorporate mindfulness techniques, which have been shown to aid in pain management among endurance athletes (Passigli et al. 2017). Environmental adaptation is equally critical, given Saudi Arabia’s distinct conditions. Extreme heat necessitates hydration strategies that go beyond general recommendations, such as electrolyte supplementation, while desert terrain demands skills for navigating uneven surfaces (Tarawneh & Chowdhury 2018).

Cultural and contextual considerations are paramount. Saudi Arabia’s climate, with summer temperatures often exceeding 45°C, poses risks of dehydration and heatstroke, which is absent in temperate regions (Tarawneh & Chowdhury 2018). Its cycling culture, still emerging, differs from Western models, with religious practices (e.g. prayer times) and language preferences (Arabic) shaping participation (Bahari & Kerari 2024). An SMP must integrate these factors, offering Arabic resources, flexible scheduling and heat-specific protocols, to ensure adherence. General guidelines, often designed for milder climates or established cycling nations, fail to address these nuances (Bahari & Kerari 2024; Masoudi et al. 2025). For example, European cycling programmes emphasise rain preparedness, which is irrelevant to Saudi Arabia’s arid environment, while neglecting desert-specific challenges such as sand hazards.

Patellofemoral pain syndrome is one of the most widespread overuse injuries among recreational cyclists and other physically active populations; however, it remains under-recognised and poorly handled worldwide (Crossley et al. 2016; Lankhorst et al. 2016). Even while exercise, education and load management have been shown to be beneficial therapies, there are currently few structured SMPs created especially for PFPS, especially in culturally diverse settings such as Saudi Arabia. Many cyclists are at risk for chronic pain, functional difficulties and decreased physical activity because of this gap, which may have an effect on their long-term health and athletic performance (Collins et al. 2022; Rathleff et al. 2013). Through the development of a culturally sensitive SMP that integrates clinical best practices and strategies for engaging patients, this research addresses an important demand and has received approval from specialists. This document presents a scalable model that could lead to the development of PFPS-specific SMPs worldwide, thereby reducing the economic, social and individual burdens associated with this widespread condition. This method is applicable beyond Saudi Arabia.

Our study employs a modified Delphi approach to identify, refine and validate a SMP for Saudi recreational cyclists. Engaging a panel of regional and international experts ensures scientific rigour and cultural relevance. The resulting framework aims to empower cyclists to manage PFPS and other risks, enhancing participation safety and enjoyment. Addressing this gap is vital as cycling continues to grow in Saudi Arabia, supporting public health goals and fostering a sustainable recreational culture.

## Research methods and design

A modified Delphi technique was employed to gather consensus from a panel of expert physiotherapists specialising in musculoskeletal dysfunction and sports injuries to develop a SMP for recreational cyclists in Saudi Arabia experiencing PFPS. The modified Delphi approach is grounded in the principle that collective expert opinions surpass individual perspectives (Brady 2015). It enables a panel of experts to collaboratively address complex issues through an iterative process. In our study, the method facilitated consensus among specialists on an SMP for recreational cyclists with PFPS in Saudi Arabia.

A panel of multidisciplinary experts in the management of PFPS, including physiotherapists, sports medicine specialists, exercise physiologists and biokineticists, were recruited based on predefined eligibility criteria. To qualify, experts had to have either published at least one research article on PFPS or possess a minimum of 10 years of experience in managing musculoskeletal conditions. Initially, experts from 10 countries (two per continent) were invited, considering their interest in cycling and its cultural relevance to Saudi Arabia. However, because of a low response rate, the recruitment focus shifted to experts available worldwide who were from Saudi Arabia, Egypt, Ghana, Nigeria and South Africa, ensuring alignment with cultural and civic perspectives. While this adjustment strengthened the contextual and cultural relevance of the panel to the Saudi setting, it may also have reduced the diversity of perspectives and potentially limited the generalisability of findings to non-regional contexts. Ultimately, 25 experts participated in Round 1 of the Delphi. Table 1 summarises their demographic details, professional positions and academic qualifications.

**TABLE 1:** Demographic characteristics of Delphi study participants across two rounds.

Variable	Round 1 (*n* = 25)	Round 2 (*n* = 19)
**Gender**		
Male	21	16
Female	4	3
**Age (years)**		
25–34	5	5
35–44	9	6
45–54	8	7
55–64	3	1
**Professional group**		
Physical Therapy	22	16
Sports Medicine	7	5
Academia	11	7
Exercise Physiology	1	1
Biokinetics	1	0
**Years of working experience with cyclists**		
< 5	12	10
5–10	2	2
11–15	6	4
16–20	1	1
> 20	4	2
**Years of working experience in managing PFPS**		
< 5	4	3
5–10	4	2
11–15	9	8
16–20	2	2
> 20	6	4

PFPS, patellofemoral pain syndrome.

### Delphi procedure

The Delphi process was initiated to develop a consensus-based SMP for recreational cyclists in Saudi Arabia experiencing PFPS. Experts, including physiotherapists, biokineticists, sports medicine specialists, academics and sports physiologists, were purposively selected and invited via email. Each invitation included an information sheet and a consent form outlining our study’s objectives, methodology and assurances of confidentiality. Upon receipt of informed consent, the modified Delphi technique was conducted over two rounds to ensure systematic and iterative refinement of the programme’s content.

### Round 1

The first round launched on 04 December 2024, with a detailed questionnaire distributed to the panel of experts. This tool was informed by foundational concepts identified through a recent systematic review (Masoudi et al. 2025) and was designed to capture expert judgements on critical components of the proposed SMP for PFPS. Using an ordinal rating scale, experts assessed various domains, including the programme’s objectives, core self-management principles, relevant outcome measures, criteria for success, and contextual and cultural considerations unique to Saudi Arabian recreational cyclists. In addition, the panel provided input on preferred pain relief strategies. In Round 1, several items achieved the predefined consensus level (≥ 70%), while others required further evaluation. Furthermore, qualitative feedback from experts highlighted several important components not included in the initial questionnaire but considered critical for an effective and context-specific self-management strategy. These suggestions informed the development of new items and the refinement of existing ones for inclusion in the Round 2 questionnaire, enhancing both the comprehensiveness and cultural relevance of the proposed programme.

### Round 2

Building on the feedback from Round 1, Round 2 of the Delphi study commenced on 27 December 2024. Experts received a revised questionnaire via Google Forms, which retained the previously validated items and incorporated new content based on suggestions from the expert panel. These additions reflected a broader scope of competencies necessary for delivering a comprehensive, patient-centred SMP for PFPS. In total, 11 new items were introduced. These included competencies in interpersonal skills, with an item assessing the ability to negotiate realistic goals with patients; professional behaviour, focusing on demonstrating cultural sensitivity when working with patients and colleagues; and reflective practice, with an item aimed at seeking and utilising feedback to enhance clinical performance. Under evidence-based practice, a new item was added to evaluate the ability to critically assess and apply research findings in clinical decision-making. The teaching and coaching domain was expanded with four items addressing patient education, the use of instructional strategies, mechanisms for delivering feedback and the promotion of self-management. Additionally, leadership and management competencies were strengthened through three items that evaluated leadership capacity, time and resource management and advocacy skills.

These enhancements aimed to ensure the final tool captured the multidimensional skills required for effective implementation of self-management strategies in physiotherapy practice.

**FIGURE 1:**
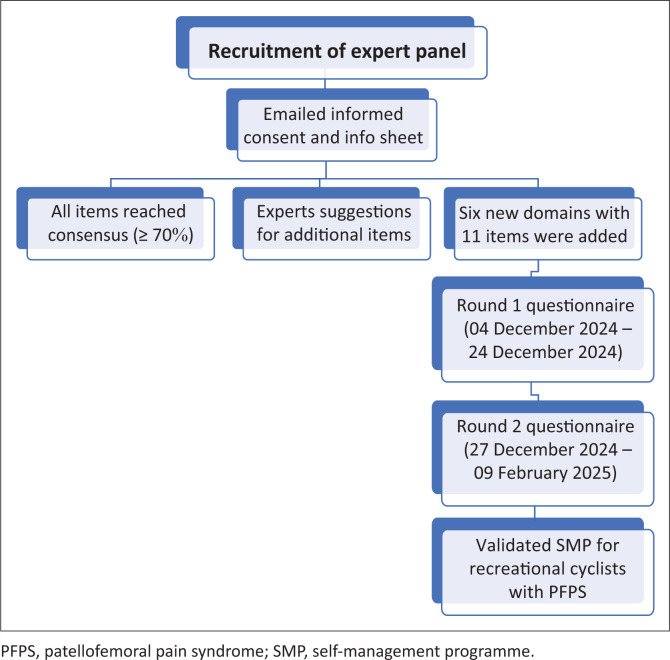
Flowchart of the modified Delphi panel process with two rounds.

Experts re-evaluated these newly introduced items using the same ordinal scale, and data collection continued until 09 February 2025. Descriptive statistics were used to determine the levels of agreement. With the achievement of consensus across all items, including those added in Round 2, a third round was deemed unnecessary. Hence, the two-round Delphi process, grounded in evidence and enriched by expert clinical insights, resulted in a validated, consensus-based SMP tailored to the cultural and clinical needs of recreational cyclists with PFPS in Saudi Arabia. Figure 1 shows the summary of the modified Delphi panel process.

### Data analysis

We exported the survey results directly from the online platform and analysed them using Microsoft Excel 19.0. We set a threshold of at least 70% of panellists agreeing or disagreeing with a statement to reach a consensus. For calculation purposes, ‘Essential’ and ‘Useful’ were grouped together as agreement, while ‘Unnecessary’ reflected disagreement. ‘Uncertain’ responses were excluded from consensus calculations. We used the categories ‘Essential, Useful, Unnecessary, and Uncertain’, and excluded ‘slightly agree’ and ‘slightly disagree’ from the overall consensus calculation. Instead, we used ‘other comments’ for exploratory questions or open-ended responses. These qualitative responses were reviewed and coded manually to identify common ideas and recurring themes. Similar suggestions were grouped together and informed the refinement or addition of items in Round 2, ensuring that cultural considerations and practical insights were incorporated into the final framework.

### Ethical considerations

An application for full ethical approval was made to the University of KwaZulu-Natal’s the Biomedical Research and Ethics Committee (BREC) on 22 August 2023. The ethics approval number is BREC/00005623/2023. To ensure informed participation, experts received a study information document and provided signed consent before participating. Confidentiality and anonymity were maintained by handling responses in accordance with established ethical guidelines, ensuring that no identifying information was disclosed. Participation was voluntary, and no financial or material incentives were offered.

## Results

A two-round modified Delphi survey was conducted to reach a consensus among experts on the components of a SMP for PFPS among recreational cyclists. Twenty-five experts completed the first round, while 19 completed the second round, resulting in a 76% retention rate. The process was iterative, allowing items with moderate support in Round 1 to be re-evaluated and refined in Round 2. Across both rounds, substantial agreement was observed, and by the end of Round 2, consensus (defined as ≥ 90% agreement) was achieved for most of the proposed items. Table 1 presents the demographic characteristics of the participating experts.

In the first round, 25 specialists participated, with a majority being male (84%) and most falling within the age range of 35–54 years. Nineteen specialists completed the second round, maintaining a comparable gender and age distribution. The panel was predominantly composed of physiotherapists, with contributions from other fields, including sports medicine, academia, exercise physiology and biokinetics. The clinical experience of the participants with cyclists varied, although nearly half indicated they had less than 5 years of experience. Detailed demographic information is outlined in Table 1.

### Objectives and principles of the self-management programme

Table 2 presents the agreed objectives and principles of the SMP. From the outset, experts strongly endorsed the central objectives of improving self-management of knee pain (92% in Round 1), providing pain education (84%), and ensuring the programme was based on scientific evidence (80%). These items did not require re-evaluation in Round 2 because consensus had already been reached. Objectives with moderate support in Round 1 achieved full consensus in Round 2.

**TABLE 2:** Objectives and principles of the self-management programme for patellofemoral pain syndrome.

Domain	Item	Round 1				Round 2			
		Essential		Useful, unnecessary and unsure		Essential anduseful		Unnecessary andunsure	
		*n*	%	*n*	%	*n*	%	*n*	%
Objectives	Based on scientific evidence to inform practice	20	80.0	5	20.0	0	0	0	0
	Developing awareness of self-postural screening to support preventive practices	-	-	-	-	19	100	-	-
	Empowering patients to manage PFPS recurrences independently	-	-	-	-	16	84.2	3	15.8
	Enabling patient-centred knee pain management	18	72.0	7	28.0	-	-	-	-
	Improving self-management of knee pain	23	92.0	2	8.0	-	-	-	-
	Improving adherence to rehabilitation programmes	17	68.0	8	32.0	19	100	-	-
	Improving patient empowerment for effective self-management of PFPS	-	-	-	-	19	100	-	-
	Including detailed preventive practices to enhance programme effectiveness	-	-	-	-	18	94.8	1	5.2
	Incorporating cycling-specific adjustments	15	60.0	10	40.0	19	100	-	-
	Pain Education	21	84.0	4	16.0	-	-	-	-
Principles	Clinical process and outcome indicators	19	76.0	6	24.0	-	-	-	-
	Collaboration with local healthcare providers	15	60.0	10	40.0	17	89.4	2	10.5
	Establishing a forum for cyclists with PFPS to promote peer support	-	-	-	-	17	89.5	2	10.5
	Evidence-based practice	21	84.0	4	16.0	-	-	-	-
	Health promotion and prevention	20	80.0	5	20.0	-	-	-	-
	Leadership and monitoring of SMP implementation	16	64.0	9	36.0	19	100	-	-
	Maximising function and independence	20	80.0	5	20.0	-	-	-	-
	Optimal communication	16	68.0	8	32.0	18	94.7	1	5.2

Note: (-) means either (1) the item already achieved consensus in Round 1 or (2) the item did not apply to Round 1 because it was generated based on expert feedback after Round 1.

SMP, self-management programme; PFPS, patellofemoral pain syndrome.

Newly introduced objectives, including empowering patients to manage PFPS recurrences and enhancing postural screening to support preventive practices, also reached consensus (≥ 95%) in the second round, as indicated in Table 2.

### Outcome measures and programme evaluation

Consensus on outcome measures and programme evaluation criteria is presented in Table 3. Pain-related outcomes were consistently prioritised, with 88% of experts in Round 1 rating reduction in pain intensity as essential, and nearly all agreeing on its importance by Round 2.

**TABLE 3:** Outcome measures and programme evaluation criteria.

Domain	Item	Round 1				Round 2			
		Essential		Useful, unnecessaryand unsure		Essential anduseful		Unnecessary and unsure	
		*n*	%	*n*	%	*n*	%	*n*	%
Programme evaluation criteria	Including detailed preventive practices to enhance programme effectiveness	-	-	-	-	18	94.8	1	5.2
Outcome measures	Optimal communication	16	64.0	8	32.0	18	94.7	1	5.2
	Leadership and monitoring SMP implementation	16	64.0	9	36.0	19	100	-	-
	Health promotion and prevention	20	80.0	5	20.0	-	-	-	-
	Maximising function and independence	20	80.0	5	20.0	-	-	-	-

Note: (-) means either (1) the item already achieved consensus in Round 1 or (2) the item did not apply to Round 1 because it was generated based on expert feedback after Round 1.

SMP, self-management programme.

Improvement in cycling performance (80%) and enhanced knee function and mobility (84%) were also strongly endorsed, highlighting the relevance of performance-based and functional outcomes for an active population. Patient satisfaction emerged as an additional indicator of programme success (76%), as indicated in Table 3.

### Enabling factors, implementation and pain relief strategies

The broader contextual factors supporting SMP implementation are summarised in Table 4. While enabling factors such as local support groups (24% in Round 1) and family or peer support (44%) initially received modest endorsement, their importance increased considerably by Round 2 (74% and 89.5%, respectively).

**TABLE 4:** Enabling factors, implementation and cultural considerations and pain relief strategies.

Domain	Item	Round 1				Round 2			
		Essential		Useful, unnecessary and unsure		Essential anduseful		Unnecessaryand unsure	
		*n*	%	*n*	%	*n*	%	*n*	%
Enablingfactors	Pain education	21	84.0	4	16.0	-	-	-	-
	Reduction in pain intensity	22	88.0	3	12.0	-	-	-	-
	Improvement in cycling performance	20	80.0	5	20.0	-	-	-	-
	Increase in knee function and mobility	21	84.0	4	16.0	-	-	-	-
Implementation and cultural considerations	Regular feedback from healthcare professionals	13	52.0	12	48.0	18	94.7	1	5.2
	Including psychological aspects in the outcome measures	-	-	-	-	18	94.7	1	5.2
	Long-term follow-up on pain management	17	68.0	8	32.0	18	94.7	1	5.2
	Use of Arabic language resources	17	68.0	8	32.0	18	94.7	1	5.2
	Sensitivity to religious practices during physical activities	17	68.0	8	32.0	17	89.5	2	10.5
Pain relief strategies	Mindfulness techniques for pain management	23	92.0	2	8.0	17	89.5	2	10.5
	Use of heat pads or cold therapy	11	44.0	14	56.0	18	94.7	1	5.2
	Self-massage techniques for the knee area	13	52.0	12	48.0	18	94.7	1	5.2
	Using self-myofascial release techniques for knee pain relief	-	-	-	-	18	94.7	1	5.2
	Incorporating hip external rotation and abduction exercises	12	48.0	13	52.0	16	84.2	3	15.8
	Utilising technology (e.g. apps, wearables) for tracking pain and progress	11	44.0	14	56.0	16	84.2	3	15.8

Note: (-) means either (1) the item already achieved consensus in Round 1 or (2) the item did not apply to Round 1 because it was generated based on expert feedback after Round 1.

The use of digital tools (apps and wearables) to track pain and progress was also supported, reflecting an openness to technology-assisted care as indicated in Table 4.

### Consensus progression across rounds

**FIGURE 2:**
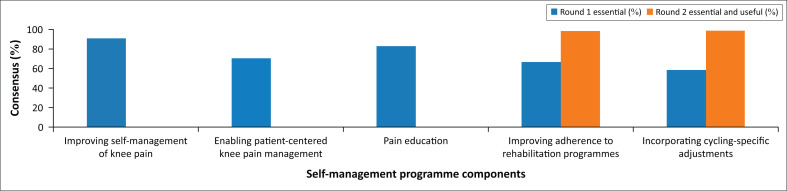
Progression of the consensus across the Modified Delphi rounds.

Figure 2 illustrates the progression of consensus across rounds. Several items that initially had only moderate endorsement in Round 1, including adherence to rehabilitation, cycling-specific modifications, online resources and local support mechanisms, reached full consensus by Round 2.

The results demonstrate the value of iterative Delphi rounds in refining expert opinion, encouraging reflection, and building agreement around items that may initially seem peripheral but gain recognition as essential with collective discussion.

## Discussion

This modified Delphi study identified expert consensus on the core components of SMP for PFPS in recreational cyclists in Saudi Arabia. The findings contribute significantly to the development of a culturally tailored, patient-centred and evidence-informed intervention framework for physiotherapists and health professionals managing PFPS. In the first round, 25 experts participated; however, six withdrew before the second round, resulting in a retention rate slightly above 75%, which is consistent with or slightly higher than rates typically reported in similar Delphi studies (Mokkink et al. 2010). This discussion expands on our study’s five major themes, which include (1) patient empowerment and education, (2) function and performance outcomes, (3) programme evaluation, (4) cultural considerations and (5) biopsychosocial pain management.

### Emphasis on patient empowerment and education

A unanimous agreement among experts on objectives such as pain education, patient-centred care and enhancing self-management reflects an evolving healthcare paradigm where patients are co-managers of their conditions. Recent studies affirm that empowering patients through structured self-management interventions improves long-term adherence and health outcomes in musculoskeletal disorders (Diener 2021; Du et al. 2011). In physiotherapy practice, this aligns with behavioural change techniques, such as motivational interviewing and shared decision-making, which enhance self-efficacy and patient engagement (Koehn & Esdaile 2008). While structured SMPs appear to improve adherence and outcomes for musculoskeletal disorders, the evidence supporting them remains somewhat limited and inconsistent, particularly in the long term (Uritani, Koda & Sugita 2021). Education that integrates pain mechanisms, biomechanics, and injury prevention has been shown to improve compliance and reduce the recurrence of injuries (Khan & Brukner 2011). Incorporating self-screening and recurrence management into the SMP reflects this preventive orientation, consistent with recent guidelines that advocate for athlete self-monitoring to reduce the risk of reinjury and enhance self-regulation (Koehn & Esdaile 2008). Similarly, Podlog et al. (2015) highlight that organised self-management initiatives that incorporate tracking symptoms and recognising early warning signs lower recurrence rates in athletes healing from musculoskeletal injuries. Participants experience enhanced self-efficacy and a quicker identification of possible reinjury.

### Integration of functional and performance outcomes

Experts in our study agreed that outcomes such as pain intensity reduction, functional knee improvements, and cycling performance enhancement should be core indicators of SMP effectiveness. This aligns with current sports physiotherapy frameworks, which prioritise return-to-performance metrics alongside pain and function (Lampros, Wiater & Tanaka 2022). In cyclists with PFPS, addressing lower limb alignment, neuromuscular imbalances, and kinetic chain deficits is essential. Previous studies have shown that individualised rehabilitation targeting hip abductor strength and dynamic stability leads to significant symptom improvement and faster return to sport (Santos et al. 2015). Emerging research emphasises that satisfaction, trust, and perceived progress are as critical to rehabilitation success as clinical outcomes (Ferreira et al. 2023). These patient-reported outcome measures (PROMs) should be integral to physiotherapy practice and service evaluation.

### Evaluation and long-term monitoring

Programme evaluation components, including adherence monitoring, regular feedback and long-term follow-up, received high endorsement from experts. These are consistent with contemporary best practices that advocate for continuous quality improvement and adaptive care pathways in physiotherapy (Adams & Neville 2020; Brewster et al. 2015; Palm & Hochmuth 2020).

Digital health tools were moderately supported but represent a growing frontier. Wearable technology, mobile apps and remote monitoring systems have shown promise in improving adherence and capturing real-time data in sports injury management (Germini et al. 2022). For example, app-based pain tracking and exercise reminders have been associated with increased programme compliance in active populations (Zhang et al. 2022). Physiotherapists should consider integrating these technologies into their workflow to facilitate hybrid models of care, especially when working with athletes or clients in remote regions.

### Cultural relevance in programme design

The panel’s emphasis on Arabic-language materials, flexible scheduling and accommodation of religious practices highlights the importance of culturally sensitive programme design. Cultural tailoring is increasingly recognised as a determinant of adherence and engagement in physiotherapy, especially in Middle Eastern contexts (Nasr 2025). Several studies have highlighted that programmes aligned with patients’ cultural values, language and daily routines are more likely to result in meaningful behaviour change and sustained health improvements (Browne et al. 2012). For Saudi recreational cyclists, sensitivity to gender norms, religious commitments and language accessibility is essential for promoting inclusion and adherence. Therefore, physiotherapists must be equipped with cross-cultural communication skills and be prepared to adapt intervention strategies to meet the cultural expectations of their patient population (O’Shaughnessy & Tilki 2007).

### Pain management and psychosocial strategies

Consensus was strong for incorporating both physical and psychological strategies for pain relief, such as strengthening exercises, mindfulness and pain neuroscience education. These strategies reflect a modern, biopsychosocial approach to chronic musculoskeletal pain, which is now considered best practice in rehabilitation (Smart 2023). Pain neuroscience education (PNE) has demonstrated efficacy in reducing pain catastrophising and improving function in individuals with PFPS and other anterior knee pain disorders (Louw et al. 2016). When combined with therapeutic exercise, mindfulness and graded exposure, it provides a comprehensive framework for addressing both physical impairments and psychological barriers. Physiotherapists are therefore encouraged to develop competencies in pain communication, stress management and basic psychological support. Even in settings where psychological services are limited, physiotherapists can incorporate simple techniques, such as breathing exercises, progressive relaxation and positive self-talk, to promote recovery (Diener 2021).

### Strengths and limitations

The Delphi method provided a rigorous and structured platform for achieving expert consensus. The high response and retention rates strengthen the reliability of the findings. Moreover, the panel’s diversity in experience and cultural awareness enhanced the breadth of perspectives captured. However, as with all Delphi studies, the reliance on expert opinion introduces potential bias. The panel size, although consistent with methodological standards, limits the generalisability of findings. In addition, the lack of empirical testing means that the recommended SMP remains a theoretical model requiring real-world validation. Future pilot studies should assess the programme’s feasibility, acceptability and clinical effectiveness among Saudi recreational cyclists.

### Implications for physiotherapy practice

Our study presents a robust, consensus-based framework that physiotherapists can utilise to develop structured SMPs for managing PFPS in active populations. The findings highlight the need for:

- Patient empowerment and shared decision-making as core tenets of care
- Performance-focused rehabilitation tailored to cycling biomechanics
- Integration of psychosocial support and cultural competency
- Continuous outcome monitoring and programme adaptation

Incorporating these elements can enhance patient outcomes, improve adherence and reduce healthcare burdens associated with recurrent PFPS. Hence, physiotherapists must evolve into culturally responsive and evidence-based practitioners, as well as interdisciplinary collaborators, to lead this transformation.

## Conclusion

In conclusion, our study provides a consensus-driven framework for an SMP tailored to recreational cyclists with PFPS in Saudi Arabia. By integrating evidence-based pain relief, cultural sensitivity and cyclist-specific outcomes, the programme addresses a critical gap in regional sports medicine. Its key contributions include combining physical and psychological strategies, embedding cultural relevance and aligning with best practice in rehabilitation. Beyond the Saudi context, the framework has potential for regional and international adaptation, particularly in cycling communities with similar cultural and sporting needs. Future research should focus on piloting this SMP in diverse clinical and community settings to evaluate its feasibility, acceptability and impact on long-term outcomes.
